# We need a NICE for global development spending

**DOI:** 10.12688/f1000research.11863.1

**Published:** 2017-07-25

**Authors:** Kalipso Chalkidou, Anthony J. Culyer, Amanda Glassman, Ryan Li

**Affiliations:** 1 Global Health and Development Group, Institute of Global Health Innovation, Imperial College London, London, SW7 2AZ, UK; 2 Institute of Health Policy, Management and Evaluation, University of Toronto, Toronto, ON, M5T 3M6, Canada; 3 Department of Economics & Related Studies, University of York, York, YO10 5DD, UK; 4 Center for Global Development, Washington, DC, 20036, USA

**Keywords:** Value for money, aid, transition, cost-effectiveness, health technology assessment, priority setting, universal coverage

## Abstract

With aid budgets shrinking in richer countries and more money for healthcare becoming available from domestic sources in poorer ones, the rhetoric of value for money or improved efficiency of aid spending is increasing. Taking healthcare as one example, we discuss the need for and potential benefits of (and obstacles to) the establishment of a national institute for aid effectiveness. In the case of the UK, such an institute would help improve development spending decisions made by DFID, the country’s aid agency, as well as by the various multilaterals, such as the Global Fund, through which British aid monies is channelled. It could and should also help countries becoming increasingly independent from aid build their own capacity to make sure their own resources go further in terms of health outcomes and more equitable distribution. Such an undertaking will not be easy given deep suspicion amongst development experts towards economists and arguments for improving efficiency. We argue that it is exactly *because* needs matter that those who make spending decisions must consider the needs not being met when a priority requires that finite resources are diverted elsewhere. These chosen unmet needs are the true costs; they are lost health. They *must* be considered, and should be minimised and must therefore be measured. Such exposition of the trade-offs of competing investment options can help inform an array of old and newer development tools, from strategic purchasing and pricing negotiations for healthcare products to performance based contracts and innovative financing tools for programmatic interventions.

## Aid is wasted

Up to 40% of healthcare spending is wasted according to the World Health Organization (WHO; ( World Health Organization, 2010)). A recent report by the Organisation for Economic Cooperation and Development puts healthcare waste – defined broadly as over-utilisation of technologies, unwarranted hospital admissions, corruption and inefficient pharmaceutical markets – at 20% ( OECD 2017). WHO analysis suggests that even the poorest or most fragile states in Sub Saharan Africa rely on external funding for less than a quarter of their total healthcare spending, and in all bar a handful the trend shows a rising domestic over foreign spending ( Soucat, 2017). By implication, a lot of healthcare aid money does not buy health, while even the world’s poorest countries increasingly finance their healthcare systems (whether wastefully or efficiently) out of extremely limited domestic resources.

None of this means that more external money for development is now unimportant, but what matters, at least as much as the amount of aid, is what Glassman (2015) calls the “priorities ditch”: a dearth of investment in governance know-how for setting spending priorities locally, and in better incentives that link aid investment to development results, in a context-sensitive and hence more effective manner ( Glassman & Chalkidou, 2012). There are lots of things donors can do, but don’t do, to fill this governance gap and to create the capacities (both internally and within countries) for wise spending, and, where countries are within reach of the Sustainable Development Goals, to smooth a transition to a world less dependent on aid.

## Filling the priorities ditch

Given today’s aid scepticism in major funding nations, credible mechanisms that actually deliver better development outcomes – whether poverty reduction, health, sanitation, nutrition, education, upholding of human rights – for the poorest and most vulnerable people must surely be our priority. The dramatic proposed cuts in the USAID budget ( Konyndyk, 2017) and the reluctant UK commitment to a 0.7% GDP aid spending target reflect public scepticism of the return-on-investment of aid. The numerous channels through which UK aid is distributed also suggest multiple objectives and a lack of strategic purpose and coordination: the Department for International Development (DFID), Foreign and Commonwealth Office, UK Trade, Research Councils, Commonwealth Development Corporation, Ministry of Defence, etc. The scepticism is shared by some sections of the UK popular media and Parliament. It ought to be possible to moderate such scepticism: a loud and clear commitment by the US and UK administrations to ensuring that money spent on aid is money well spent, good value for those giving and for those receiving. Emphasis on analysis, evidence and performance, rather than evidence-free advocacy and non-performance-linked targets, may be a win-win for both donors and recipients of aid ( Chalkidou *et al.*, 2017). But this will require a much sharper focus within major global donors and – most importantly – in-country training in the necessary analytical skills and in-country infrastructure for recipients, so that competent agencies and ministerial departments are created and sustained ( Li *et al.*, 2017).

To make the case, it is no use relying solely on emotional case-by-case appeals to “doing the right thing” or on a parade of advocacy-friendly global statistics. Instead, before any funds are committed, donor countries should insist that the necessary capacity in recipient countries is brought into being to ensure that only cost-effective investments are considered in the first place. Mere effectiveness is not enough. The only relevant kind of cost-effectiveness is that determined by the social values and development objectives (better health, better education, and so on) in the country, the budget it proposes, and the associated realistic cost-effectiveness threshold, above which not a single significant investment should be made without very compelling reasons. A serious and sustained institutionalised effort is needed to analyse and publish every significant aid programme’s return-on-investment, monitoring and evaluating it both during implementation and in the longer run. Ian Mitchell of the Center for Global Development proposes that any bilateral aid programme above £10m should be disallowed unless this kind of analysis has been done and the proposed investment passes the relevant tests ( Mitchell, 2017). He also proposes the establishment of NIDE, a National Institute for Development Effectiveness, to play this role on behalf of the UK.

## NIDE: NICE for aid?

Modelled along the lines of the National Institute for Health and Care Excellence (NICE), NIDE would be an independent public body accountable to the Government, and whose function is to assess the value-for-money of overseas development assistance on behalf of DFID and other relevant agencies. NIDE would evaluate the value-for-money of investing in aid not only for bilateral programmes, but also for monies spent through multilateral agencies – in health aid, such agencies include the Global Fund for AIDS, TB and Malaria, UNITAID, and Gavi – where the opportunities for efficiencies, given the bulk purchasing of commodities on behalf of large part of the world, are significant. Indeed, Her Majesty’s Government made a start in this direction with last September’s Performance Agreement with the Global Fund: “ *Through our membership of the Board of the Global Fund, the UK will work to strengthen independent advice and scrutiny of the Global Fund to ensure that it is following best practice in seeking value for money.”* ( Department for International Development and The Global Fund to Fight Aids, Tuberculosis and Malaria, 2016). ** What are missing, and what a NICE-for-aid could provide, are value-for-money indicators and valid, reliable processes for measuring and reporting against them.

NIDE would also determine two other key factors in conjunction with government departments of recipient countries. One is the nature of criteria other than cost-effectiveness to be deployed to inform investment decisions. For health investments, evident candidates include the benefits of financial protection that the investments may enable and the contribution the investment would make to reducing the worst inequalities in health. The other factor is the cost-effectiveness threshold suitable for each client country, set so that when the country eventually absorbs the costs of whatever investments the aid has established (for instance, vaccination programmes), those costs are affordable with domestic resources, and in the case of health not the absurdly generous thresholds inherited from past WHO priority-setting ( Culyer, 2016; Revill *et al.*, 2014). NIDE would need also to play a key role in the creation of training and capacity-building programmes – targeting a broad spectrum of stakeholders ranging policymakers, technical officers, researchers, and the like – and preferably such programmes located in recipient countries and preferably ones that also train trainers, thereby avoiding as far as possible excessive ongoing dependence on the UK and other high-income countries ( Li *et al.*, 2017).

The “NICE-for-aid” idea is not new for public policy, at least not in the UK. The What Works Centres claim “a world first” for any government to have *“…taken a national approach to prioritising the use of evidence in decision-making.”* ( Ruiz & Breckon, 2014). What Works Centres span health (NICE is part of the What Works network), crime, education and ageing, but do not (yet) cover development. The NIDE proposal simply extends this world innovation in public policy to overseas development assistance. This ought to win over domestic aid sceptics by championing strong governance and institutions in recipient countries alongside rigorous value-for-money assessments – assessments using world class skills, which the UK prides itself on and has in abundance ( Hasan *et al.*, 2015).

NIDE must not be a patronising or culturally imposing exercise. The BMGF, Rockefeller and DFID already fund the international Decision Support Initiative (iDSI) based at Imperial College. This is a multinational, multiprofessional and multidisciplinary initiative aiming at improving health resource allocation decisions in low and middle income countries (LMICs) using evidence of comparative clinical and cost-effectiveness ( Chalkidou, *et al.*, 2017). It operates by strengthening local capacities for decision-making and priority-setting, by promoting best practice in analytical and statistical methods, and by sharing experiences and results actively through its expanding network. iDSI works with the national governments or national health insurers of India, South Africa, Ghana, Indonesia, Vietnam and China to improve value-for-money in healthcare investments. The World Bank is coordinating a Joint Learning Network for Universal Health Coverage, which is somewhat broader in scope and has recently launched an efficiency collaborative with similar objectives to those of iDSI. And whilst demand from governments for such support is less evident in low income economies, funding channels, such as the Global Fund and UNITAID, can play this role. In its market shaping strategy, the Global Fund commits to: *“…proactively engage with recipients to share relevant analyses and information about likely product costs and comparative health technology assessments…the GF Secretariat...will connect recipients with these resources to inform country-driven health technology assessment. Engaging in this process can also be an opportunity to build country capacity for health technology assessment and how to incorporate this into product selection decisions.”* ( Kanpirom *et al.*, 2017).

## NICE for aid: Setting it up

Setting up a NICE-for-aid would hardly be an easy task. There are challenges in setting the scope (health, education, other areas of social policy, commodities or services, etc.) and in defining (even inventing) the methods to be followed for evaluating aid investment projects both *ex ante,* and perhaps *ex post* in monitoring and evaluation, where perhaps much of the ODA Research Council money could be channelled.

On *scope*, healthcare spending is an obvious starting point given the global drive for universal health coverage – Target 3.8 of the Sustainable Development Goals. Within healthcare, NIDE should seek a broad agreement on the value of evidence and the principles according to which evidence would be appraised; on the principles for determining value-for-money (economic efficiency); on suitable outcome measures and their appropriate uses; and on the principles to be used in assessing ethical and distributional issues and their integration into Health Technology Assessment ( Chalkidou *et al.*, 2016). NIDE should also make an inventory of the evidence-based policy expertise – essentially expertise in evidence generation, governance, and policy – that the UK has after the Cochrane Collaboration, more than 15 years’ experience of NICE, and two decades of Health Technology Assessment. NIDE should also liaise with the National Institute of Health Research and the Research Councils to develop a research programme that addressed important unanswered but researchable questions – ones whose answering would enhance NIDE’s effectiveness. **

On *methods*, starting with articulating a Reference Case, that is an agreed “gold standard” for conducting and reporting economic analyses, to drive better economic evaluations makes sense. iDSI already has a Reference Case for health economic evaluations in low- and middle-income countries ( Wilkinson *et al.*, 2016). A Reference Case would have two main tasks: first to list the essential characteristics of competent research and research reporting, and second to list those specific contextual matters that can be resolved only in-country. NIDE could usefully revisit the current DFID definition of Value for Money (which has little economic content), the 4Es framework, which confusingly comprises 3 Es and a fourth CE for cost-effectiveness - but inexplicably discusses neither opportunity costs nor allocative efficiency ( Department for International Development, 2011).

NIDE should avoid taking a blinkered approach on the scope of economic and epidemiological analyses. They provide useful tools for looking at countries transitioning away from aid, to inform, for instance, appropriate co-financing levels to maximise return-on-investment. Recent work has advanced the field of efficiency and performance measurement of whole healthcare systems ( Smith & Yip, 2016), moving well beyond the NICE approach of assessing only individual pharmaceutical products to include the analysis of delivery platforms, health system strengthening interventions and human resource constraints ( van Baal *et al.*, 2017; Morton *et al.*, 2016). This is not easy – a recent attempt at dealing with a DFID programmatic intervention on maternal and child health in Nigeria as a technology whose value could be assessed compared to the next best thing, proved to be incredibly hard ( Jones, 2017). Hard - but not impossible. Hard - but in fact essential if such investments are not to waste aid money. At the very least, the Nigeria study threw up areas where more research, empirical or methodological, or data collection exercises, or investment in skills were needed.

*Data* is another important prerequisite for systematically assessing the value for money of aid interventions. Systematic assessment is not only valuable for the substantive implications it has for particular policy choices; it also identifies data gaps and focuses attention on future data collection priorities amongst types of information that would make a difference in future investment decisions. Data on unit costs (price transparency is one important element of this) and resource use (how much does it cost the National Health Insurance of Ghana to treat a stroke?), incidence (how much of the full cost falls on private individuals), individual and social preferences (reflecting local cultural and religious realities), data on risks (incidence data for most conditions for example are absent for most SSA countries) and effect sizes (inevitably based on pragmatic research carried out in in-country context; ( BOLDER Research Group, 2016)), treatment of residual uncertainty (for example, where data are absent and resort has to be had to modelling and “expert opinion”). Such a targeted, decision needs-driven approach to data generation, ideally through systems’ own routine mechanisms, need not necessarily be complemented by monster demographic health surveys, but it would make decisions easier to make, the reasons for them clearer, and decision makers more accountable to their populations and their donors. NICE has had that very effect in the National Health Service (NHS): costing data on technologies and services (like reference costs and later diagnosis-related groups) became more readily available as NICE used them for informing real investment decisions.

## The opposition?

NIDE is bound to provoke hostility. If it does things wrong, this will be deserved. But let us review a few grounds that would *not* be good grounds for complaint.

It will be said that costs ought not to be considered when setting health care priorities ( Loewy, 1980). Only need matters. The charge is deeply wrong because it is inconsistent. It is exactly *because* needs matter that NIDE must consider the needs not being met when a priority requires that resources go elsewhere. These chosen unmet needs are the true costs. They are lost health. They *must* be considered, and should be minimised (and must therefore be measured).

It will be said that people’s willingness to pay ought not to determine the priorities in a public health insurance system because of the huge inequalities in abilities to pay in most LMICs. Indeed, individual willingness ought not to be the determinant. But explicit collective willingness to pay is essential, this is most handily expressed by a cost-effectiveness threshold and NIDE will have to help countries to decide what this should be. The WHO’s 1–3 times GDP per capita for health may well have caused more harm than good, until its quiet withdrawal by WHO sometime in 2016/17, for being overly generous and not nearly country specific enough to inform meaningful local spending decisions ( Revill *et al.*, 2014; Woods *et al.*, 2016). Here is an issue over which one size definitely does not fit all. In particular, setting a threshold that is too high ensures that it becomes impossible to implement a cost-effective programme of care. It ensures that services recommended on its basis are unaffordable.

Some will rail against economics and economists, attributing to them an indifference to inequities and uncritical worship of market solutions. Major figures in global development have attributed inequalities in healthcare outcomes and access to economics and economics. “Value for money”, “sustainability”, a “minimum healthcare package” and “limited resources” are deemed to be too un-aspirational and depriving the poor in developing countries from services they need, in the name of efficiency (Paul Farmer, Who Lives and Who Dies; ( Farmer, 2015)). Senior WHO officials recently declared any efforts to value benefits of the latest expensive on-patent drugs, or “the value of life”, simply “unfeasible”. And economists are accused of “institutionalising inequality” and being collectively against “access free at the point of delivery”, which “kills the soul of an economist” ( Richard Horton, The Atlantic, 2012) ( Meyer, 2013). There are of course some economists who (still) believe in healthcare markets, consumer supremacy and prices as the best proxies for people’s preferences, all ill-suited to healthcare and most public policy, but these economists are not amongst those who published the Economists’ Declaration on UHC ( Summers, 2015). True, NIDE must choose its economists well!

None of these objections should stop the UK from improving the effectiveness of aid spending, starting with its own investment and building on the firm principle that we seek to maximise the impact of our aid money on the health of the people we choose to help. If DFID were to create a NICE-for-aid it would at a stroke improve purchasers’ ability to buy effectively at all levels: from the way DFID spends its own money, through bilateral country programmes or organisations such as Global Fund, UNITAID and Gavi, as well as the WHO and the World Bank, to influencing how the likes of the Global Fund or World Bank trust funds contract directly with product manufacturers or pass money on to countries for them to buy from service providers or product manufacturers. Such an approach would give new meaning to “strategic purchasing” or “evidence-informed procurement”. It could also inform upstream investment decisions made by the like of DFID’s CDC, the UK private investment arm, active in South Asia and Sub Saharan Africa.

## What would it cost?

It has been estimated that the HTA programme in the UK has much more than paid for itself. Implementing just ten reports of the hundreds produced, for only one year, and conservatively assuming a yield of just 12% of the total benefit assumed in the HTA analyses, generates enough value to cover the running of the whole HTA programme for 20 years ( Guthrie *et al.*, 2016). That is surely a return-on-investment to die for. NICE, one of the most expensive of HTA agencies in the world, costs less than 0.05% of the NHS budget. A review of priority setting institutions around the world found that those countries who do have them, spend less than 1/1000 of their resources on them ( Glassman *et al.*, 2012). Spending less than 0.1% of the total health care budget on deciding how to spend the remaining 99.9% more wisely, improving outcomes and access, and building the needed local technical and administrative capacity in the process, is surely the definition of a good investment.

## …and without a NICE-for-aid?

What is the alternative? The risk is that the UK’s (and possibly USA’s) aid spending becomes increasingly vulnerable to sceptics, leading to further budget cuts, and further fragmentation between spending channels across government departments. The “business as usual” approach would rule: serving short term objectives rather than maximising long term impact on reducing poverty and jeopardising progress toward valuable development goals, such as universal health coverage. Without the work a NIDE could do, countries transitioning away from aid will become more at risk of regressing back to being aid-dependent states, the local intelligent capacity for making good decisions will remain unbuilt, and the world risks losing hard won gains towards sustainable global development ( Glassman & Temin, 2016). It can be done. Let’s do it!
