# Supplementary material for: We need a NICE for global development spending
Source: F1000Res. 2017 Jul 25;6:1223. [Version 1] doi: 10.12688/f1000research.11863.1 (PMC5558097; doi:10.12688/f1000research.11863.1)
Supplement: Peer review history

# Peer review: Referee response for version 1

**DOI:** 10.5256/f1000research.12819.r24491

**Published:** 2017-08-15

**Authors:** Daniel A. Ollendorf

**Affiliations:** 1 Institute for Clinical and Economic Review, Boston, MA, USA

This is a thought-provoking piece advocating for a quasi-governmental body that uses evidence-based principles to ensure the efficient, cost-effective, and equitable use of donor funds for global health programming directed at low-and-middle income countries. The authors have a wealth of experience in managing the use of international aid monies in the developing world, and been heavily involved in training and implementation of health technology methods and decision support techniques in those settings.  

 The authors' work with iDSI is instructive, but readers will benefit from specific examples of the change brought about the organization's activities. For example, has an evaluation been performed to compare decision-making processes before and after an iDSI "intervention" in India, Ghana, etc.? I've been recently made aware of an "affordable cancer drugs list" for India - was this an iDSI effort and how has its performance contrasted with prior approaches?

 Specific comments are also provided below:

- Abstract: just a minor grammatical correction. "...is channeled" should be "are channeled."
- Pg 3, column 1, paragraph 3: Proposed cuts to USAID and UK commitments certainly reflect skepticism around return-on-investment, but they also likely reflect the presence of nationalist politics, and this should be mentioned.
- Pg 3, column 2, paragraph 3: This seems an opportune place to introduce budgetary impact analysis as a companion effort to cost-effectiveness and related efforts. While successful empiric approaches to setting WTP thresholds will explicitly consider health budgets, BIA can be flexibly defined to address contingencies common in the developing world (e.g., regime change, changes in aid vs. domestic funding balance, etc.)
- Pg 4, column 1, paragraph 4: Scope challenges are appropriately noted, and I agree that health spending is a natural starting point. However, it would be useful to see some context around spending challenges across sectors in the developing vs. developed world.
- Page 5, column 1, paragraph 1: It's very difficult for me disentangle the very real data needs mentioned from the reality of trying to collect data in these settings. An example of a data need that was simply and efficiently addressed in an LMIC would be helpful. The example of NICE's impact is less relevant for me given the resources available in the UK.
- Page 5, column 2, paragraph 3: It may be premature to do this, but ROI is both about paying for and sustaining NIDE's activities AND reducing wasteful aid spending. Some estimate of wasteful spending in the UK, and what percentage reduction would essentially pay for NIDE and increase access to health services in some number of countries would be helpful, even if back-of-the envelope.

I have read this submission. I believe that I have an appropriate level of expertise to confirm that it is of an acceptable scientific standard.

# Peer review: Referee response for version 1

**DOI:** 10.5256/f1000research.12819.r24492

**Published:** 2017-08-07

**Authors:** Samantha A. Hollingworth

**Affiliations:** 1 School Pharmacy, University of Queensland, Brisbane, QLD, Australia

This interesting paper argues the case for an agency potentially named the National Institute for Development Effectiveness (NIDE) - like a NICE for global health spending by bilateral and multilateral aid agencies. The authors are well placed to propose this agency given their wealth of experience in this area. The paper outlines the need, the proposed mechanism, aspects of establishment, and requirements (e.g. methods and data). The authors pre-emptively outline some potential arguments opposing NIDE and posit the alternative – what would happen if there was no NIDE? They also outline the anticipated costs.

 The authors note that this is not a new idea for the UK (p4) and mention the UK Government’s What Works initiative (some aspects of social policy) and NICE (health technology assessment). It might be useful to hear more about what other countries or multilateral agencies may have done in this area especially with regard to establishment, methods, and data sources

 It appears that health technology assessment would be a major role for NIDE but it may be worth also considering the costs of scale up and implementing interventions (and not only budget impact analysis).

 The authors anticipate some hostility. They mention several aspects but this is based on selected challenges once NIDE would be established. The authors may want to comment on one or two reasons that some may oppose even the establishment of NIDE. There may be several (and possibly competing) imperatives for some particular ‘aid’ projects funded by government(s). Concerning the negative arguments about ‘economics’, one can point to NICE itself for the benefits of ‘economics’!

 The authors helpfully outline what NIDE might cost but it would be instructive to know the amount of money spent on aid – at least by the UK. Indeed, the authors hint at the excellent anticipated return on investment for NIDE - allocation efficiency for aid!

 Some particular comments:

- P3, col 2 - The authors pose some good information requirements. [“What are missing, and what a NICE-for-aid could provide, are value-for-money indicators and valid, reliable processes for measuring and reporting against them.”] but it would be helpful to have some more detail on these vital components.
- P4 col 1 - Could mention that the UK government funds the ‘What works’ initiative (for non-UK audiences).
- P4 col 2 - Please define the three Es from the 3E framework.
- P5 col 1 - It is unclear about the use of the term ‘incidence’ (presumably of disease or condition) with its example of (how much of the full cost falls on private individuals)’ which seems related to funding sources.

I have read this submission. I believe that I have an appropriate level of expertise to confirm that it is of an acceptable scientific standard.
